# Policy to Decrease Low Birth Weight in Indonesia: Who Should Be the Target?

**DOI:** 10.3390/nu15020465

**Published:** 2023-01-16

**Authors:** Ratna Dwi Wulandari, Agung Dwi Laksono, Ratu Matahari

**Affiliations:** 1 Faculty of Public Health, Universitas Airlangga, Surabaya 60115, Indonesia; 2 The Airlangga Centre for Health Policy (ACeHAP), Surabaya 60115, Indonesia; 3 National Research and Innovation Agency, the Republic of Indonesia, Jakarta 10340, Indonesia; 4 Faculty of Public Health, Ahmad Dahlan University, Yogyakarta 55164, Indonesia

**Keywords:** low birth weight, maternal and child health, maternal health, public health, nutrition, health policy

## Abstract

The study aimed to analyze the target of the policy to decrease low birth weight (LBW) in Indonesia. This cross-sectional study used a sample of live births in last five years preceding the survey of birth weight. Data collection took place from July to September 2017. The weighted sample size was 17,848 participants. The variables analyzed included residence, age, marital status, education, employment, parity, and wealth. The study employed binary logistic regression in the final stage to determine the target of policy regarding LBW. The results showed that women in urban areas were 1.200 times more likely to deliver babies with LBW than women in rural areas. All age groups were less likely to deliver babies with LBW than those aged 45–49. The study also found all marital statuses had a lower likelihood of providing babies with LBW than those who had never been in a marriage. Women of all education levels had a greater risk of giving birth to babies with LBW than women with higher education levels. Unemployed women had 1.033 times more chances of delivering babies with LBW than employed women. Primiparous women were 1.132 times more likely to give birth to babies with LBW than multiparous women. Overall, the women in all wealth status categories had a higher probability of delivering babies with LBW than the wealthiest groups. The study concluded that policymakers should target women who live in urban areas, are old, have never been married, have low education, and are unemployed, primiparous, and poor to decrease LBW cases in Indonesia.

## 1. Introduction

A newborn should have an average body weight of ≥2500 kg. The baby is categorized as having a low birth weight if its weight is <2500 g (1500–2499 g). Birth weight is crucial for infant development because it is associated with infant mortality [1,2]. A study states that birth weight is an essential indirect indicator of maternal nutrition and a predictive predictor of potential infant mortality and malnutrition. Due to these issues, researchers nowadays measure birth weight as an indicator of health. Babies born with low birth weight are at high risk of neonatal mortality. They are also at risk of experiencing stunting, delays in brain nerve development, and other diseases at this stage of development [3,4]. Another impact of LBW is increasing neonatal deaths globally by around 60–80% [5].

The incidence of LBW babies is more than 20 million births each year globally. Overall, the global prevalence shows that the LBW rate in developed regions was 27.2%, compared to 17.3% in Asia, 5.6% in Central Asia, and 27.2% in Southern Asia [3]. Low birth weight became the focus of the World Health Organization and United Nations Children’s Fund (UNICEF) in 1992, and then the WHO targeted ending malnutrition by 2025 globally. Based on the WHO’s data, 15% of babies worldwide experienced LBW, and more than half were babies born in Asia [6]. Risk factors related to the incidence of LBW include socioeconomic and maternal characteristics. Socioeconomics deals with the type of residence, occupation type, parents’ educational status, and wealth index [7]. At the same time, maternal factors include preterm delivery, a history of low birth weight, maternal age, height, hemoglobin level, lack of iron supplementation, and frequency of Antenatal Care (ANC) visits [8]. LBW is also related to exclusive breastfeeding behavior, which significantly supports a baby’s growth and development in the first two years [4].

A previous study showed that other factors that influence the incidence of LBW, including cultural practices, are crucial to be addressed by health workers in providing maternal and child health care services. Indonesia is rich in artistic methods for treating diseases and caring for mothers and newborns, which can cause maternal and infant mortality [9]. The trend of LBW babies in Indonesia in 2018 was 6.2%. This figure had decreased by 4% from 2013, when the rate was 10.2% [10]. Although Indonesia has experienced a decrease in the number of LBW babies, this percentage has not met the target reduction of cases of 3% per year [10]. The situation is also related to the number of stunting incidents resulting from LBW. Indonesia is a developing country ranked fifth for the highest LBW babies out of 88 countries worldwide [11]. The incidence of stunting in Indonesia reached 37.8% in 2015 and 31% in 2018. This decrease in percentage has not yet attained the annual reduction target of 7.3% [12].

Based on the factors related to LBW, the Indonesian government strives and is committed to accelerating nutrition levels through the ‘Scaling Up Nutrition (SUN)’ program. The government includes long-term, medium-term, and short-term development plans regulated by the law. In the long-term development plan (2005–2025), the Indonesian government focuses on the first 1000 days of life through the fulfilment of nutrition for the womb until the baby is two. The government carries out cross-sectoral synergies in concrete steps to improve food production, processing, and consumption that meet nutritional needs. Such actions increase nutrition awareness, promotion, education regarding physical and health activities, and access to quality science- and technology-based nutrition services [13,14]. Furthermore, the Indonesian government has initiated the *‘Desa Siaga’* or Alert Village program since 1994. It is a community engagement program that seeks to help pregnant women by maximizing the resources available, such as access to transportation, costs, and social support in their living areas [15].

The most critical part of implementing the policy for LBW prevention is educating pregnant women and young women (adolescents) who will prepare for pregnancy. Health workers can attempt to minimize beliefs in cultural behavior that can endanger pregnant women’s and babies’ health and safety and improve the quality of baby feeding, especially exclusive breastfeeding [16,17]. Advocacy and dissemination of information to the community by healthcare workers will help empower knowledge of LBW prevention [9]. Based on the background, the study aimed to analyze the target of the policy to decrease LBW cases in Indonesia.

## 2. Materials and Methods

### 2.1. Data Source and Study Design

This cross-sectional study analyzed secondary data from the 2017 Indonesia Demographic and Health Survey (IDHS). It used a sample of live births in the last five years preceding the survey, comprising birth weight, written records, and maternal memories. The author used the ‘IDIR71FL_Individual Recode’ files.

The stratified two-stage sampling design used in the 2017 IDHS was as follows: Stage 1: selecting several census blocks systematically proportional to size probability with the number of households resulting from the 2010 population census listing. In this example, an implicit stratification procedure based on urban and rural regions was used for sorting census blocks based on the wealth index category of the 2010 population census data. Stage 2 picks 25 ordinary households in each census block based on updating the households in each census block [18]. The 2017 IDHS collected data from July to September 2017. The 2017 IDHS investigated the babies’ mothers as respondents among live births within the last five years. Around 94% of mothers reported their babies’ birth weight; the study selected as many as 17,848 participants as the sample in the final process.

### 2.2. Variables

The study used LBW as a dependent variable. It defined LBW as a birth weight of fewer than 2500 g regardless of gestational age [18]. Apart from LBW as the dependent variable, the study analyzed seven independent variables: the type of residence, age group, marital status, education level, employment status, parity, and wealth status.

Following the Indonesian Central Bureau of Statistics, the study divided residences into urban and rural categories. The study split ages into seven groups: 15–19, 20–24, 25–29, 30–34, 35–39, 40–44, and 45–49 years. Education level, the last certificate owned, was categorized into four categories: no education, primary, secondary, and higher. Marital status had three categories: never in marriage, married/living with a partner, and divorced/widowed.

Meanwhile, this study divided employment status into two categories: unemployed and employed. Parity, the acknowledgment of the number of live babies ever born, was studied in three categories, namely primiparous (<2), multiparous (2–4), and grand multiparous (>4).

The 2017 IDHS described wealth status as the socioeconomic quintile in a household. It was related to household income as seen from the styles and prices of furniture, such as televisions, bicycles, motorcycles, and household products, including drinking water supplies, bathroom amenities, and flooring materials. The 2017 IDHS determined the value of this variable by using the principal component analysis in the report. Every household’s score for the national wealth quintiles was then grouped into the same five classes, accounting for 20% of the population [10]. They were the poorest (quintile 1), poorer (quintile 2), middle (quintile 3), richer (quintile 4), and richest (quintile 5) [19,20].

### 2.3. Data Analysis

The study employed the bivariate test to analyze all data investigated at the first stage. In the bivariate analysis, the researchers used the chi-square test. Before conducting a binary logistic regression test, the study performed a co-linearity test to ensure no multicollinearity symptoms existed between the independent variables. The study used binary logistic regression to determine the policy’s target at the final stage. The author processed all statistical analyses in SPSS 26 software.

The study used ArcGIS 10.3 to map the percentage distribution of LBW cases by provinces in Indonesia (ESRI Inc., Redlands, CA, USA). The study collected the shapefile of administrative boundary polygons from the Indonesian Bureau of Statistics.

### 2.4. Ethical Approval

The study used secondary data from the 2017 IDHS for a materials analysis. The survey removed the identities of all respondents from the 2017 IDHS dataset. Participants in this study signed written consent forms, and the children’s parents or guardians gave their consent (under 16 years). The author has obtained permission to use data for this study through the website https://dhsprogram.com (accessed on 1 November 2020).

The 2017 IDHS adheres to the Standard D.H.S. survey protocol under the Demographic and Health Surveys Program (DHS-7), which has been approved by ICF International’s Institutional Review Board and was previously reviewed and approved by the ORC Macro IRB in 2002. DHS surveys that adhere to the Standard are classified as DHS-7 program-approved, and the approval document is attached. ICF International’s Institutional Review Board followed the US Department of Health and Human Services requirements for the “Protection of Human Subjects” (45 CFR 46).

## 3. Results

**Figure 1:**
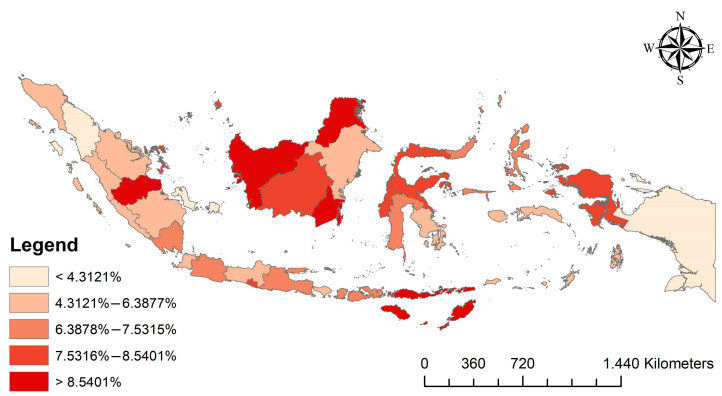
Distribution of LBW percentage by the province in Indonesia, 2017.

The national average percentage of LBW was 6.981% (95% CI 6.607–7.355%). At least 18 out of the 34 provinces in Indonesia have LBW rates above the national average. Figure 1 shows the percentage distribution of LBW cases by the provinces in Indonesia. The areas with a high percentage of LBW cases tended to be in the Northern Central parts of Indonesia.

Table 1 presents the descriptive analysis of the LBW cases in Indonesia. The percentage is in the column percentage. Based on residence, women in urban areas tended to give birth to LBW babies more, and women in the 25–29 age group broadly delivered LBW babies more. In addition to these variables, married women or women who lived with partners tended to give birth to LBW babies more.

In terms of education, women with secondary education were more likely to deliver LBW babies. Unemployed women and multiparous women tended to give birth to LBW babies. Moreover, wealthier women were more likely to have LBW babies.

The collinearity test result indicated no symptoms of multicollinearity between the independent variables tested. The tolerance value of all variables was greater than 0.10. Simultaneously, the variance inflation factor (VIF) value for all independent variables was less than 10.00. Then, referring to the basis of decision-making in the multicollinearity test, the study concluded that there was no indication of a strong relationship between the independent variables in the regression model.

According to maternal education level, mothers with only primary school education and less ruled in both nutritional status categories. Based on maternal age, stunted children under the age of five have mothers with an average age slightly older than normal children under five years.

Table 2 displays the binary logistic regression results for LBW cases. The study referenced the category “LBW = No” at this final stage. This analysis indicated that women in rural areas are less likely to give birth to LBW babies in Indonesia. Women living in urban areas are 1.200 times more likely to provide LBW babies than those in rural areas (AOR: 1.200; 95% CI: 1.200–1.200).

While women in all age group categories, except the 45–49 age group, are less likely to give birth to LBW babies. Moreover, pregnant women in all marital status categories, except those who have never been married, are less likely to give birth to LBW babies.

Women in all education level categories showed a higher probability of giving birth to LBW babies than women with higher education levels. Concerning education level, unemployed women are 1.033 times more likely to deliver LBW babies than those employed (AOR: 1.033; 95% CI: 1.032–1.033). This analysis showed that unemployment is a risk factor for women giving birth to LBW babies in Indonesia.

Regarding parity, primiparous women are 1.132 times more likely to labor LBW babies than women with many children (AOR: 1.132; 95% CI: 1.132–1.132). The information indicated that being primiparous is a risk factor for pregnant women giving birth to LBW babies in Indonesia.

Finally, Table 2 informs us that the poorest women are 1.111 times more likely to give birth to LBW babies than the most prosperous women (AOR: 1.111; 95% CI: 1.111–1.111). Women of poorer wealth status are 1.181 times more likely to deliver LBW babies than the most affluent women (AOR: 1.181; 95% CI: 1.181–1.181). Women in the middle wealth status group have 1.067 times more chances of giving birth to LBW babies than the most affluent women (AOR: 1.067; 95% CI: 1.067–1.067). Besides, wealthier women are 1.079 times more likely to give birth to LBW babies than the most prosperous women (AOR: 1.079; 95% CI: 1.079–1.079). This analysis implied that women in all wealth status categories except the most decadent have a higher probability of giving birth to LBW babies.

## 4. Discussion

Nationally, the average percentage of LBW cases, at 6.981% in 2017, was below the target of the National Medium-Term Development Plan in 2015–2019. However, at least 10 out of 34 provinces in Indonesia did not experience a lower percentage of LBW cases below this target. Besides, the Indonesian government recorded 18 regions with a prevalence of LBW cases above the national average [10]. This study informs us that women living in urban areas were more likely to give birth to LBW babies in Indonesia. This finding aligns with the previous research in Afghanistan, reporting that mothers who lived in rural residences were 0.3 times less likely to give birth to LBW babies than those in urban areas [21]. This condition is likely related to higher pollution in urban than rural areas of Indonesia. A previous study found that higher paternal prenatal concentrations of mono-benzyl phthalate and mono-carboxyisononyl phthalate were linked to a 40% and 53% increase in the incidence of LBW in infants who were born spontaneously [22]. Moreover, previous studies have indicated that a higher pollution level was associated with a higher incidence of LBW [23,24].

This finding contradicts several previous studies. Studies in Ethiopia and Iran provided different conclusions. Mothers in rural Ethiopia and Iran were 1.39 and 1.4 times more likely to give birth to LBW babies than those in urban areas [25]. Another study in Sidama Zone, South Ethiopia, used public hospitals as the research setting for newborns. It found that mothers in rural areas were 3.51 times more likely to give birth to LBW patients than those in urban areas [26].

Based on the age group, the result showed women in all age group categories except the 45–49 age group had a lower likelihood of giving birth to LBW babies. Previous studies in Jordan and Ethiopia also indicated that age influenced LBW incidence [25,27]. A study in Germany reported different results, finding that teenage mothers had higher rates of depression during pregnancy than mothers of older generations. The research indicated that this factor puts adolescent mothers at risk of giving birth to LBW babies [28].

Women in all marital status categories, except those who had never married, had a lower chance of giving birth to LBW babies. An earlier study in Sub-Saharan Africa also reported marital status was associated with LBW babies. Divorced or widowed mothers were more likely to deliver LBW babies than married mothers [29]. Having a partner is a protective factor for women, and wives become a place to share psychological and financial burdens [30]. Besides, mothers in all education level categories had a higher probability of giving birth to LBW babies than women with higher education levels. A study in Jordan and Ethiopia also found similar results: a higher education level was a protective factor for mothers not giving birth to LBW babies [25]. Meanwhile, previous studies in Indonesia showed that pregnant women with primary school education were 2.154 times more likely to give birth to LBW babies (AOR 2.154, 95% CI 1.399–3.315; *p* = 0.004) [1]. Because teaching is a motivating opportunity to live healthily, education is good for your health. Women who receive an education will have more opportunities to develop their abilities and lead healthy lives [31]. Higher-educated women were more likely to experience a better pregnancy and to experience a lower probability of delivering LBW kids [16,32]. A better education level allows mothers to better understand actions needed to provide the best output for themselves and their children [16,33,34]. Many studies have reported that better education is a strong determinant of better production in the health sector [35,36,37,38]. Instead, poor education is a barrier to producing quality performance in the health sector [8,32,39]. Unemployment was deemed a risk factor for women giving birth to LBW babies. A previous study in hospital settings in the Ambata-Tembaro zone, Southern Ethiopia, found that unemployed mothers could be 5.4 times more likely to deliver LBW babies than employed mothers [40]. Another study at a large European maternity hospital found similar effects of a multivariable analysis: unemployed women or homemakers increased adjusted odds ratios for LBW [41]. This condition was possibly related to employment and wealth status. Unemployed women are among low-income families [42].

Primiparous women had a higher probability of giving birth to LBW babies than women with many children. A study in Brazil also reported that primiparous mothers had up to 1.62 times the chance of delivering LBW babies than mothers with many children [43]. Moreover, studies in Sub-Saharan Africa and China also provided similar results, indicating that multiparity was associated with reduced cases of LBW [29]. Research in Southern Africa informed different findings that mothers with more than three births were 1.5 times more likely to give birth to LBW babies [40].

Moreover, this study revealed that women in all wealth status categories, compared to the wealthiest, had a higher probability of giving birth to LBW babies. An earlier study analyzing secondary data from the 2016 Ethiopia Demographic and Health Survey in Ethiopia also reported similar results with the incidence of LBW being dominant among the poor [44]. Poor households have limitations in providing food for all family members, including pregnant women. For pregnant women, the mother’s nutritional status is an indicator of the adequacy of food and nutrition. Better wealth status is related to the availability of food in the family and thus is closely related to food intake during pregnancy. Lack of food availability in terms of quantity and quality. This is related to the increasing need for macronutrients and micronutrients in pregnant women and their importance for the mother and fetus. Therefore, a better wealth status can reduce the incidence of LBW [45]. In terms of food insecurity and consumption, a previous study described acute and chronic malnutrition. Reducing malnutrition in children could be done through prenatal measures and supplementary food for children during food insecurity [45].

### Study Limitation

The 2017 IDHS derived the data from a stratified multi-stage sampling procedure. The sampling method allows for an unequal probability of selection.

This current study analyzed secondary data limited to variables reported in the IDHS. It excluded several variables known as determinants of LBW incidence in previous studies. Some were antenatal care during pregnancy [15,45], smoke pollution [46], wanting a later child [25], pregnancy interval and gestational age of <37 weeks at birth [47], hypertension, mid-upper arm circumference [26], maternal height [48], hemoglobin levels [49], and intimate partner violence during pregnancy [50].

Additionally, this study employed a quantitative approach and thus did not uncover the social and cultural phenomena related to LBW regarding family and children value [51,52,53], pregnancy value, and dietary restrictions in pregnant women [54], and a patriarchal social structure in Indonesia that places women subordinate to men [55].

## 5. Conclusions

Based on the results, policymakers should target pregnant women who live in urban areas, are old, have never been in a marriage, have low education, and are unemployed, primiparous, and poor to decrease LBW cases in Indonesia. The policymaker should target prospective mothers with a higher risk of having LBW babies.

## Figures and Tables

**Table 1:** The descriptive analysis of LBW in Indonesia, 2017.

Variables	Low Birth Weight		*p*-Value
	No (n = 16,602)	Yes (n = 1246)	
Type of residence			<0.001
Urban	48.3%	50.9%	
Rural	51.7%	49.1%	
Age group			<0.001
15–19	2.3%	2.9%	
20–24	16.3%	17.7%	
25–29	25.7%	25.2%	
30–34	26.4%	23.1%	
35–39	19.6%	21.5%	
40–44	8.0%	7.6%	
45–49	1.7%	2.0%	
Marital status			<0.001
Never in union	0.1%	0.2%	
Married/Living with a partner	97.2%	96.0%	
Divorced/Widowed	2.7%	3.9%	
Education Level			<0.001
No education	1.2%	1.0%	
Primary	25.6%	29.4%	
Secondary	57.6%	56.8%	
Higher	15.6%	12.8%	
Employment status			<0.001
Unemployed	55.0%	56.6%	
Employed	45.0%	43.4%	
Parity			<0.001
Primiparous	29.3%	31.5%	
Multiparous	70.7%	68.5%	
Wealth status			<0.001
Poorest	20.6%	21.1%	
Poorer	20.0%	21.8%	
Middle	20.1%	20.0%	
Richer	20.2%	20.1%	
Richest	19.1%	17.0%	

**Table 2:** The result of binary logistic regression on LBW in Indonesia, 2017.

Predictor	Low Birth Weight			
	*p*-Value	AOR	95% CI	
			Lower Bound	Upper Bound
Residence: Urban	<0.001	1.200	1.200	1.200
Residence: Rural (ref.)	-	-	-	-
Age: 15–19	<0.001	0.932	0.932	0.933
Age: 20–24	<0.001	0.870	0.870	0.871
Age: 25–29	<0.001	0.837	0.836	0.837
Age: 30–34	<0.001	0.774	0.774	0.774
Age: 35–39	<0.001	0.958	0.957	0.958
Age: 40–44	<0.001	0.813	0.813	0.813
Age: 45–49 (ref.)	-	-	-	-
Marital: Never in union (ref.)	-	-	-	-
Marital: Married/Living with a partner	<0.001	0.399	0.399	0.400
Marital: Divorced/Widowed	<0.001	0.550	0.550	0.551
Education: No education	<0.001	1.018	1.018	1.019
Education: Primary	<0.001	1.361	1.360	1.361
Education: Secondary	<0.001	1.146	1.145	1.146
Education: Higher (ref.)	-	-	-	-
Employment: Unemployed	<0.001	1.033	1.032	1.033
Employment: Employed (ref.)	-	-	-	-
Parity: Primiparous	<0.001	1.132	1.132	1.132
Parity: Multiparous (ref.)	-	-	-	-
Wealth: Poorest	<0.001	1.111	1.111	1.111
Wealth: Poorer	<0.001	1.181	1.181	1.181
Wealth: Middle	<0.001	1.067	1.067	1.067
Wealth: Richer	<0.001	1.079	1.079	1.079
Wealth: Richest (ref.)	-	-	-	-

AOR: adjusted odds ratio; LBW: low birth weight.

## Data Availability

The author cannot publicly share the data because a third party and the authors who own the data do not have permission to share the data. The 2017 IDHS data set requested from the ICF (‘data set of childbearing age women’) is available from the ICF contact via https://dhsprogram.com (accessed on 1 November 2020) for researchers who meet the criteria for access to confidential data.
